# Supplementary material for: Autophagy deficiency promotes triple-negative breast cancer resistance to T cell-mediated cytotoxicity by blocking tenascin-C degradation
Source: Nat Commun. 2020 Jul 30;11:3806. doi: 10.1038/s41467-020-17395-y (PMC7393512; doi:10.1038/s41467-020-17395-y)
Supplement: Supplementary file 1 — Supplementary Information [file 41467_2020_17395_MOESM1_ESM.pdf]

Supplementary Information

Autophagy Deficiency Promotes Triple-Negative Breast Cancer Resistance to T Cell-Mediated Cytotoxicity by Blocking Tenascin-C Degradation

Li et al.

Supplementary Methods

Immunofluorescence assay

For cell staining, the cells seeded on Glass Bottom culture dishes. For Immunofluorescence staining, the samples were stained with primary antibodies against TNC (ab108930, Abcam) and p62 (sc-28359, Santa Cruz) diluted in 4% BSA at 4°C overnight. The cells were washed, and followed by a fluorescently labeled secondary antibody Alexa Fluor 488- and Alexa Fluor 594-conjugated antibodies against mouse or rabbit IgG (Thermo Scientific), and developed with DAPI. Confocal images were examined using a microscope (Olympus). Colocalization studies were performed using ImageJ software (National Institutes of Health).

MTT Assay

The cells were seeded in 96-well plate (Falcon). Cell viability was determined by MTT assay. Briefly, MTT was added to each well for another 4 h at 37°C. After that, MTT solution was removed and replaced with 150 ul DMSO. Absorbance values with a test wavelength of 570 nm and a reference wavelength of 650 nm was read by SpectraMax Plus 384 (MD).

Immunocytochemistry staining assay

For Immunocytochemistry staining, the samples were stained with primary antibodies against TNC (GTX12298, Gentex) diluted in 4% BSA at 4°C overnight. The cells were washed, and followed by HRP polymer conjugated secondary antibody for 2.5 min and developed with diamino-benzidine solution (ZSGB-Bio). Nuclei were counterstained with hematoxylin. Image acquisition was performed using a Nikon camera and software.

Quantitative Real-Time PCR

Total RNA was isolated by TRIzol according to the manufacturer's instructions. And reverse transcription was performed using a PrimeScript™ RT Reagent Kit with gDNA Eraser (RR047D, Takara). Quantitative Real-Time PCR (qRT-PCR) was conducted using ChamQ SYBR qPCR Green Master Mix (Q311-03, Vazyme Biotech Co.)[1], and run with a Light Cycler 480 instrument (Roche Diagnostics). The relative

amount of target gene mRNA was normalized to GAPDH. All qRT-PCR reactions were done in triplicates. These primers for TNC (sense: 5'-TGCACATAGTGAAA AACAATACCCG-3', antisense: 5'-GCACGTCTTTGATGCCGTAG-3') were designed to generate a fragment of 180bp.

TUNEL staining Assay

For detection of apoptosis in the tissue sections, the terminal deoxynucleotidyl transferase dUTP nick end-labeling (TUNEL) assay was performed. TUNEL Assay Kit (Cat#C1088) was purchased from Beyotime Biotechnology. To clarify the source of TUNEL signals, we further performed double immunofluorescent staining for EpCAM (a marker for epithelial cell) or CD45 (a marker for leukocyte). After Tunel staining, the samples were then further stained with primary antibodies against EpCAM (sc-66020 , Sata Cruz) or CD45 (ab33923, Abcam) diluted in 4% BSA at 4℃ overnight. The cells were washed, and followed by a fluorescently labeled secondary antibody Alexa Fluor 647-conjugated antibody, and developed with DAPI. Confocal images were examined using a microscope (Olympus).

Clinical data

26 advanced melanoma patients data were retrieved from Gene Expression Omnibus (GSE78220). All patients were treated with either pembrolizumab or nivolumab as the anti-PD-1 therapy for their metastatic melanoma. Objective response to anti-PD-1 therapy was based on investigator-assessed immune-related Response Evaluation Criteria in Solid Tumours (irRECIST)[2]. Responding pretreatment tumours (n=13) were derived from patients who displayed complete or partial responses in response to anti-PD-1 therapy. Non-responding tumours (n=13) were derived from patients who had progressive disease. TNC expression >= lower quartile and Beclin1 expression < upper quartile were assigned as "TNC high and Beclin1 low" group, while the remaining were categorized as "Other" group. There was no significant difference in age, gender, stage, and other treatment history between the two groups.

Supplementary Figures

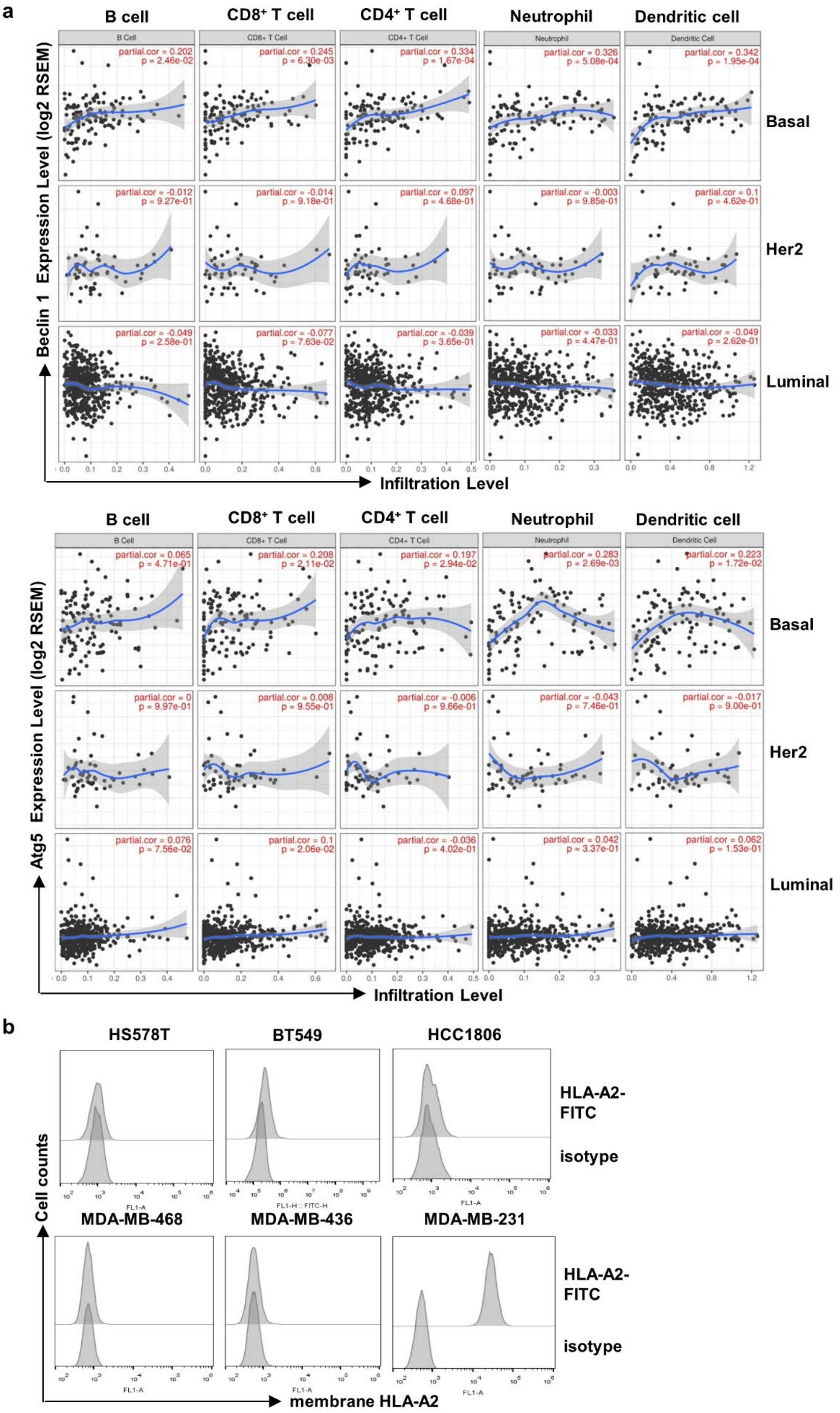

Supplementary Fig. 1 Autophagy-related genes might be involved in the tumour immune response. (a) Purity-corrected partial Spearman correlation and statistical significance of the correlation between Atg5/Beclin 1 expression and TIIC subsets (B cells, CD4 T cells, CD8 T cells, neutrophils, and dendritic cells) using the Tumor Immune Estimation Resource (TIMER 1.0) (https://cistrome.shinyapps.io/timer/). The grey shaded area represented the confidence interval of correlation coefficient. **(b)** TNBC cell lines were stained with HLA-A2-FITC antibody and analyzed by flow cytometry. The data are representative of three independent experiments.

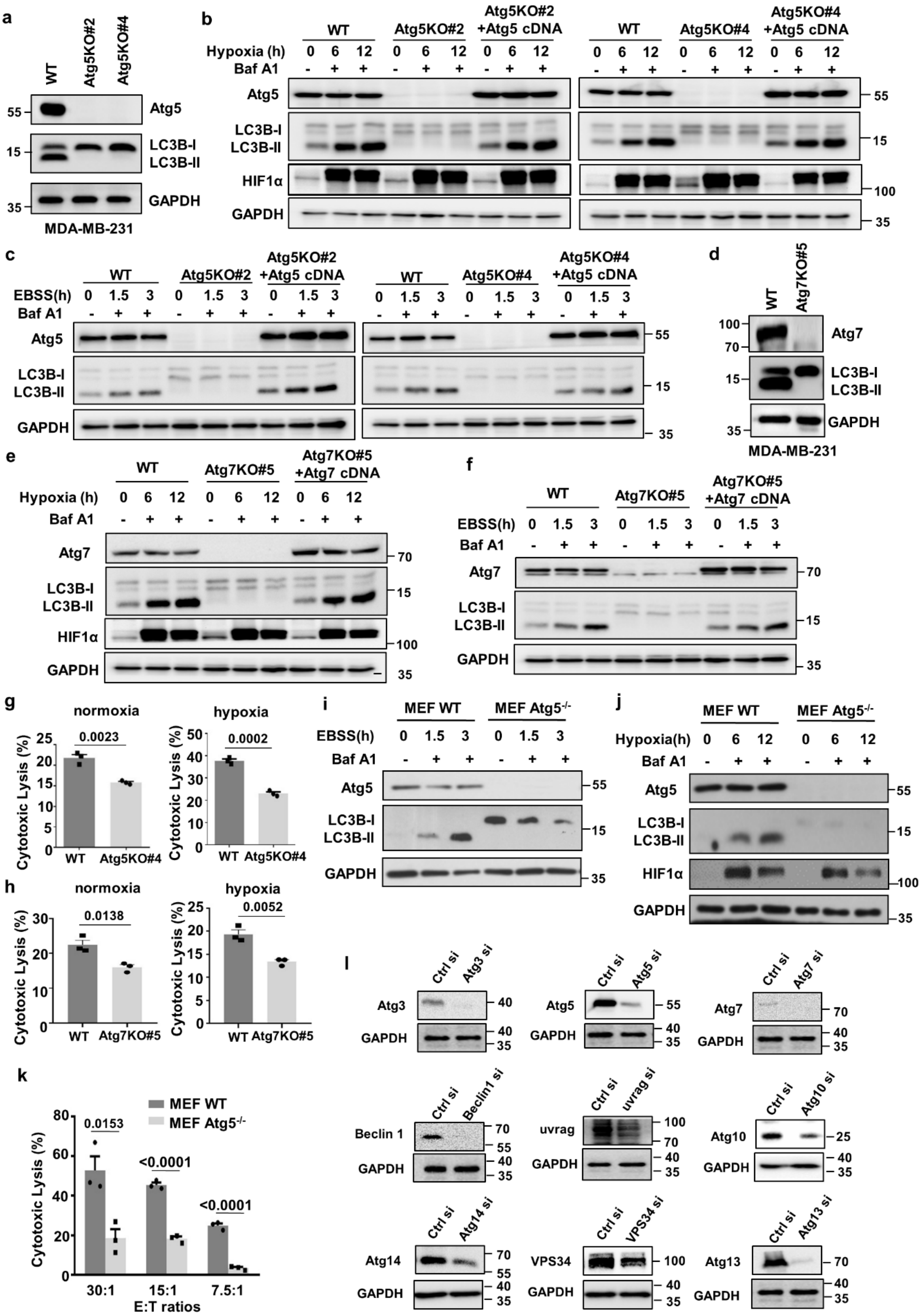
a
WT
Atg5KO#2
Atg5KO#4
Atg5
LC3B-I
LC3B-II
GAPDH
MDA-MB-231
b
WT
Atg5KO#2
Atg5KO#2 +Atg5 cDNA
Atg5KO#4
Atg5KO#4 +Atg5 cDNA
Hypoxia (h)
Baf A1
HIF1α
c
EBSS(h)
d
Atg7KO#5
Atg7
e
Atg7KO#5 +Atg7 cDNA
f
g
normoxia
hypoxia
Cytotoxic Lysis (%)
0.0023
0.0002
Atg5KO#4
h
0.0138
0.0052
i
MEF WT
MEF Atg5-/-
j
Hypoxia(h)
k
80
60
40
20
0
0.0153
<0.0001
30:1
15:1
7.5:1
E:T ratios
l
Ctrl si
Atg3 si
Atg5 si
Atg7 si
Beclin1 si
uvrag si
Atg10 si
Atg14 si
VPS34 si
Atg13 si
Atg3
Beclin 1
uvrag
Atg10
Atg14
VPS34
Atg13

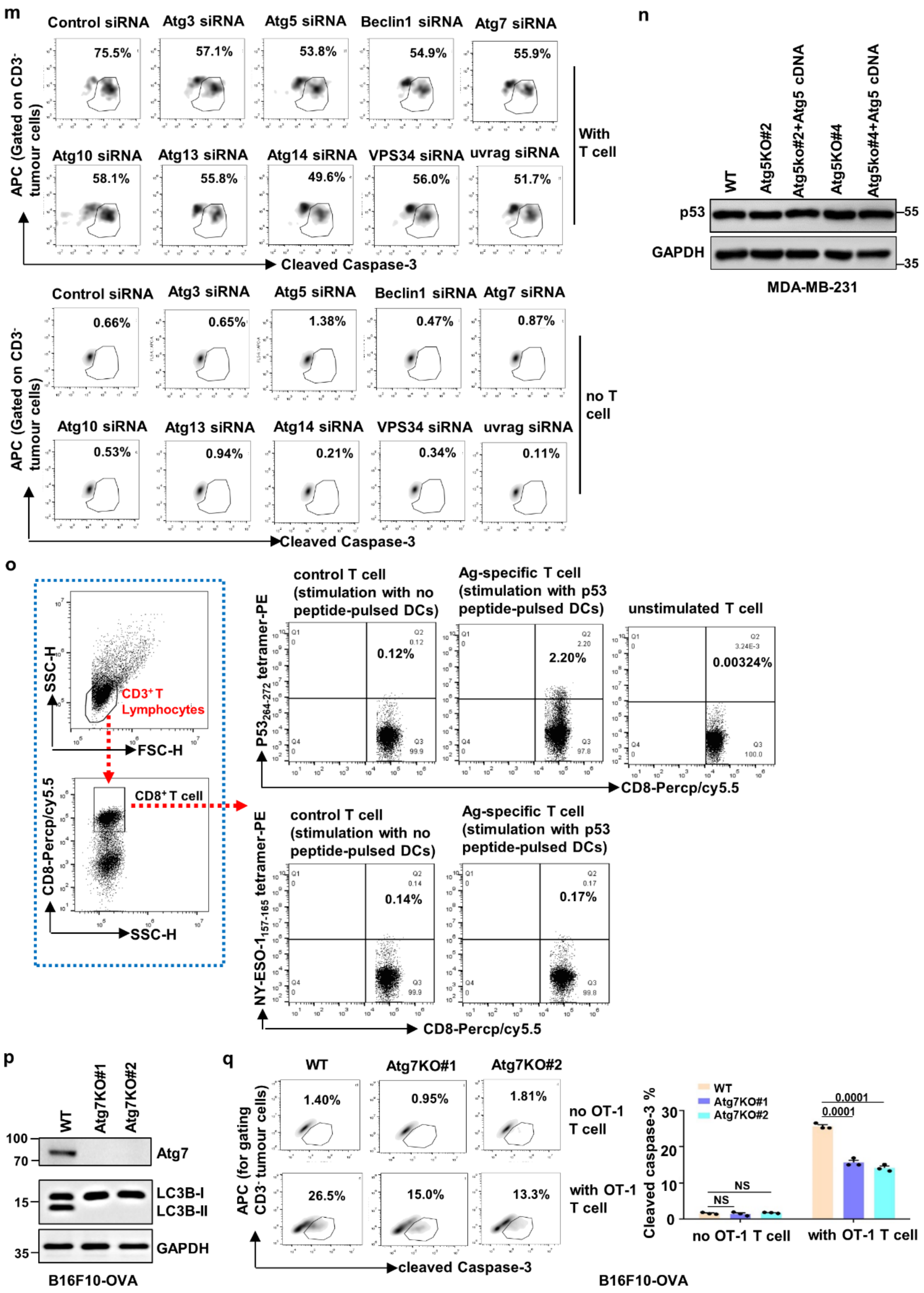

Supplementary Fig. 2 Autophagy deficiency reduces T cell-mediated tumour killing *in vitro*. **(a)** Effect of Atg5 knockout in MDA-MB-231 cells using CRISPR-Cas9 technology. **(b, c)** The indicated MDA-MB-231 cells were exposed to hypoxic conditions (1% O_2) (b) or EBSS(c) for different durations in the presence

of 100nM BafA1. **(d)** Effect of Atg7 knockout in MDA-MB-231 cells using CRISPR-Cas9 technology. **(e, f)** The indicated MDA-MB-231 cells were exposed to hypoxic conditions (1% O_2) (e) or EBSS(f) for different durations in the presence of 100nM BafA1. **(g, h)** Percent cytotoxicity was assayed by measuring the release of LDH. The indicated MDA-MB-231 cells were co-cultured with CD3/CD28 -activated human T lymphocyte cells exposed to normoxic or hypoxic conditions (1% O_2). **(i, j)** WT and Atg5$^{-/-}$ MEF cells were exposed to EBSS(i) or hypoxic conditions (1% O_2) (j) for different durations in the presence of 100nM BafA1. **(k)** CD3/CD28-activated mouse T lymphocyte cells from the spleen of BALB/c mice and the indicated MEF cells were co-cultured. Cytotoxicity was determined using an LDH assay at different effector: target (E:T) cell ratios. **(l, m)** MDA-MB-231 cells were transiently transfected with siRNA for 48 h. Then the cells were co-cultured with CD3/CD28-activated human T lymphocyte cells. The knockdown efficiency was analyzed by immunoblotting (l). Representative dot plots of the cleavage of caspase-3 in tumour cells measured by flow cytometry (m). **(n)** The expression of P53 in the indicated MDA-MB-231 cell lines. **(o)** Autologous purified T cells were cocultured with p53 peptide-pulsed DCs, followed by stained with p53$_{264–272}$ tetramer and anti-CD8. NY-ESO-1$_{157-165}$ tetramer was used as a control tetramer staining. The numbers in the upper right quadrants (Q2) of dot plots indicate the frequency of p53$_{264-272}$ tetramer^{+}CD8^{+} T or NY-ESO-1$_{157-165}$ tetramer^{+}CD8^{+} T cells. Unstimulated T cells were from PBMC of the same HLA-A2^{+} healthy donor. **(p)** Effect of Atg7 knockout in B16F10-OVA cells using CRISPR-Cas9 technology. **(q)** The indicated B16F10-OVA cells were treated with IFNγ for 24h, then cocultured with OT-1 CD8^{+} T cells isolated from spleen of OT-I TCR transgenic mice. Left, representative dot plots of the cleavage of caspase-3 in tumour cells. Right, percentage of cleaved caspase-3^{+} tumour cells. Error bars represented mean±SEM, n=3biological independent samples. The *P* value in **g, h, k** was determined by unpaired two-tailed Student's t test. The *P* value in **q** was determined by one-way ANOVA with Dunnett's multiple comparisons test, no adjustments were made for multiple comparisons. All data are representative of three independent experiments.

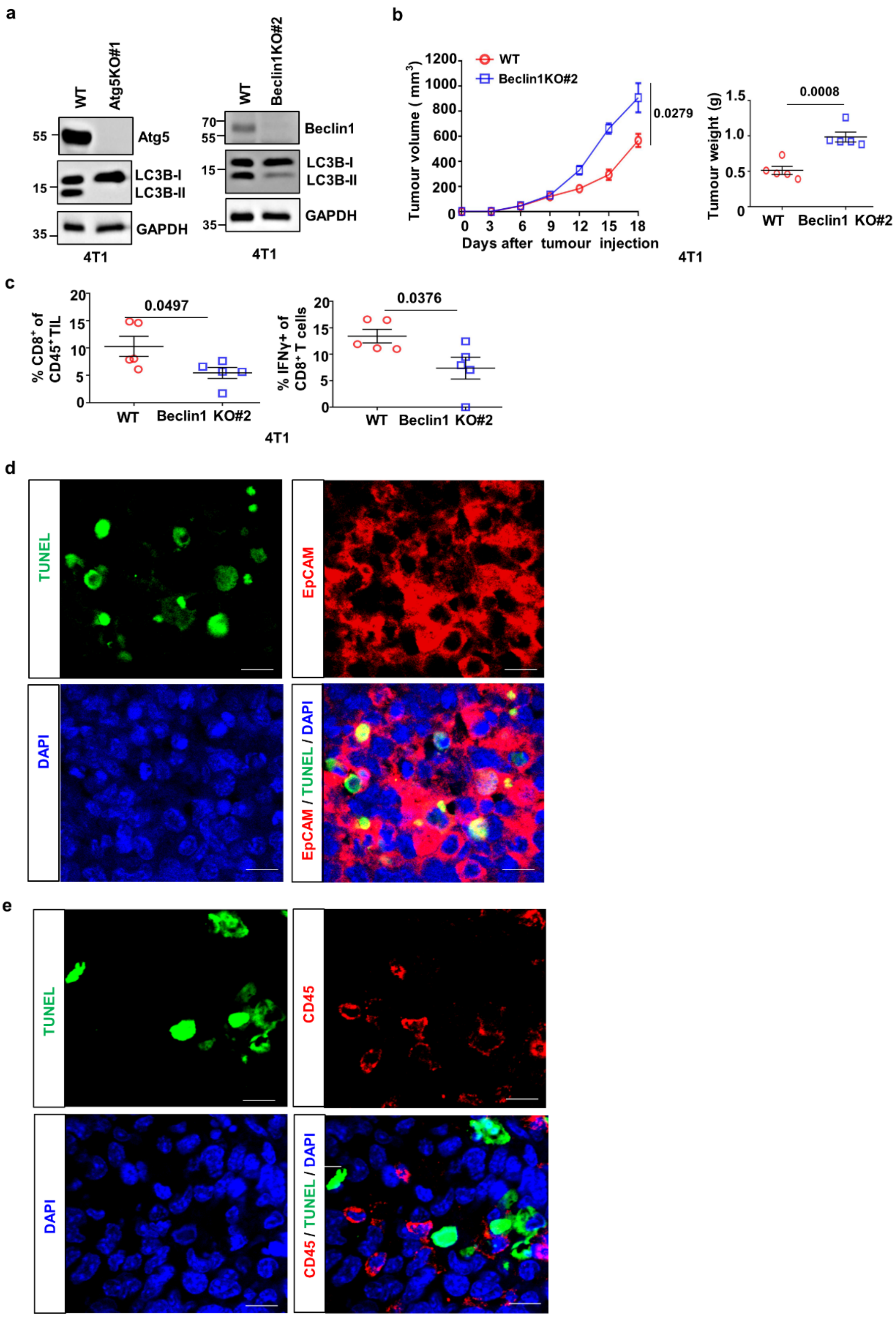

Supplementary Fig. 3 Autophagy deficiency reduces T cell-mediated tumour killing *in vivo*. **(a)** Effect of Atg5 and Beclin1 knockout in 4T1 cells using CRISPR-Cas9 technology. **(b, c)** Tumour growth of mouse 4T1-WT or 4T1-Beclin 1 KO cells in BALB/c mice (n=5 mice per group). Tumor volumes (left, b), and tumor

weights from experiment on autopsy on day 18(right, b) were calculated. FACS analysis of $CD45^{+}CD8^{+}$ and $IFN\gamma^{+}$ in $CD45^{+}CD8^{+}$ T cell populations from the isolated TILs. The percentage of TILs for each group were calculated (c). **(d)** Immunofluorescent double staining for TUNEL and EpCAM in the same section from anti-PDL1-treated 4T1-WT groups. **(e)** Immunofluorescent double staining for TUNEL and CD45 in the same section from anti-PDL1-treated 4T1-WT groups. Scar bar, 10um. Error bars represented mean± SEM, n=5 mice per group. The *P* value in **b, c** was determined by a two-tailed unpaired Student's t test. Results are representative of two independent experiments.

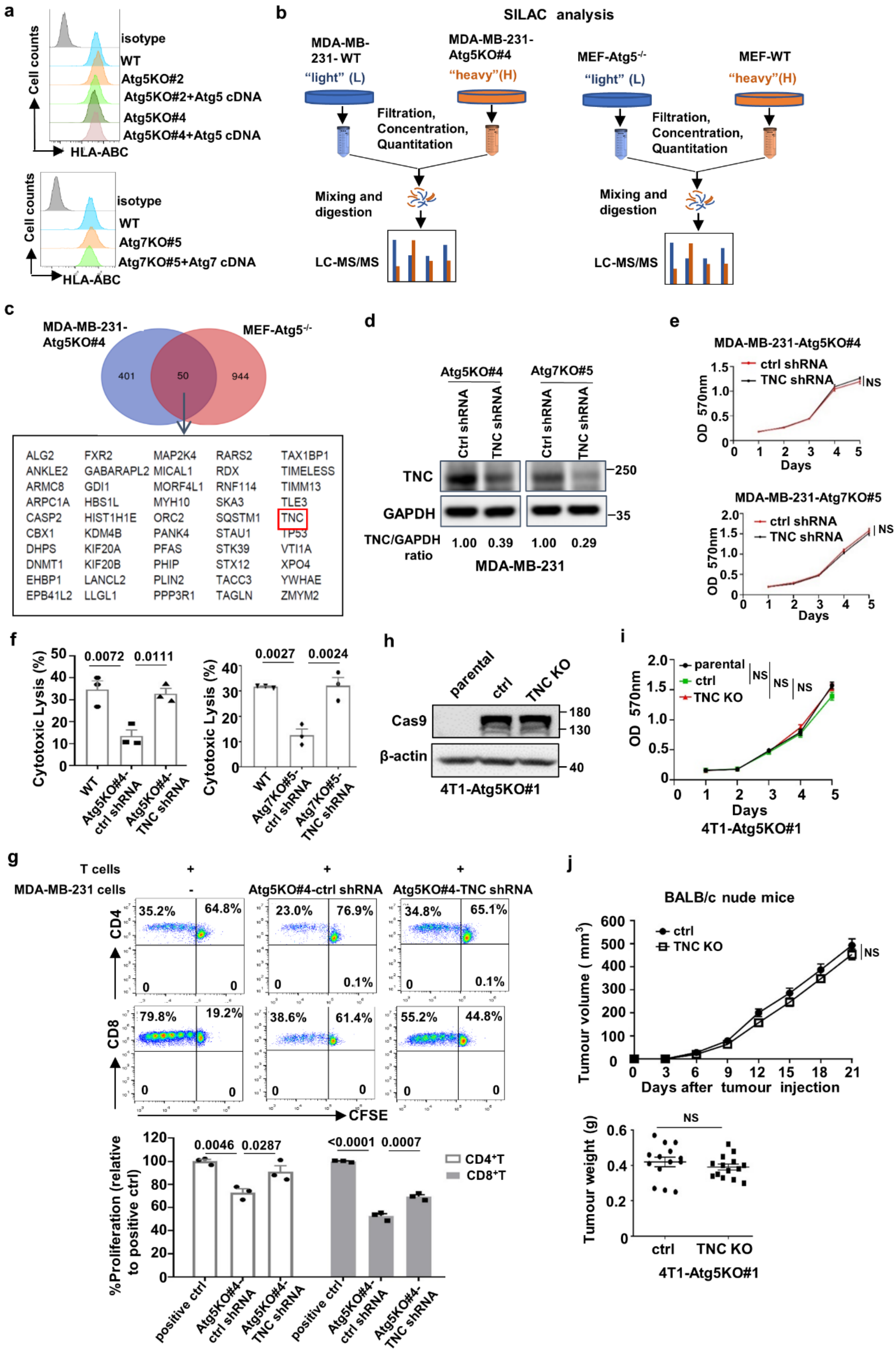

Supplementary Fig. 4 TNC is involved in autophagy deficiency-mediated immunosuppression. (a) The indicated MDA-MB-231 cells were subjected to FACS

analysis for cell surface HLA-ABC expression. **(b)** Schematic overview of quantitative stable isotope labelling by amino acids in cell culture. **(c)** Venn diagrams showing commonly up-regulated proteins in MDA-MB-231-Atg5KO#4 cells and MEF Atg5$^{-/-}$ cells. **(d)** The effect of TNC expression after the indicated MDA-MB-231 cell lines were stably transfected with TNC shRNA plasmid. **(e)** Growth curves of the indicated MDA-MB-231 cell lines transduced with shRNA targeting TNC over a 5-day period by MTT assay (n=4 biological independent samples). **(f)** The indicated MDA-MB-231 cell lines were co-cultured with CD3/CD28-activated human T lymphocyte cells. Cytotoxicity was determined using an LDH assay (n=3 biological independent samples). **(g)** Upper, representative dot plots of *in vitro* proliferation of CD4$^+$ T and CD8$^+$ T measured by FACS as CFSE dilution after 5 days, respectively, of stimulation with anti-CD3 beads alone (positive control) or in the presence of the indicated irradiated MDA-MB-231 cells (1:10 ratio) were added in coculture. Bottom, percentage of proliferating CD4$^+$ and CD8$^+$ T (n=3 biological independent samples). **(h)** The expression of Cas9 in the indicated 4T1-Atg5KO#1 cell lines. **(i)** Growth curves of the indicated 4T1-Atg5KO#1 cell lines over a 5-day period by MTT assay (n=3 biological independent samples). **(j)** Tumor growth of indicated mouse 4T1-Atg5KO#1 cells in BALB/c SCID mice. Tumor volumes were calculated (n=14 mice per group) (upper), and tumor weights from experiment on autopsy on day 21(bottom). Error bars represented mean± SEM. The *P* value in **e**, **j** was determined by a two-tailed unpaired Student's t test. The *P* value in **f**, **g**, **i** was determined by one-way ANOVA with Tukey's multiple comparisons test, no adjustments were made for multiple comparisons. NS, no significance. All data are representative of three independent experiments.

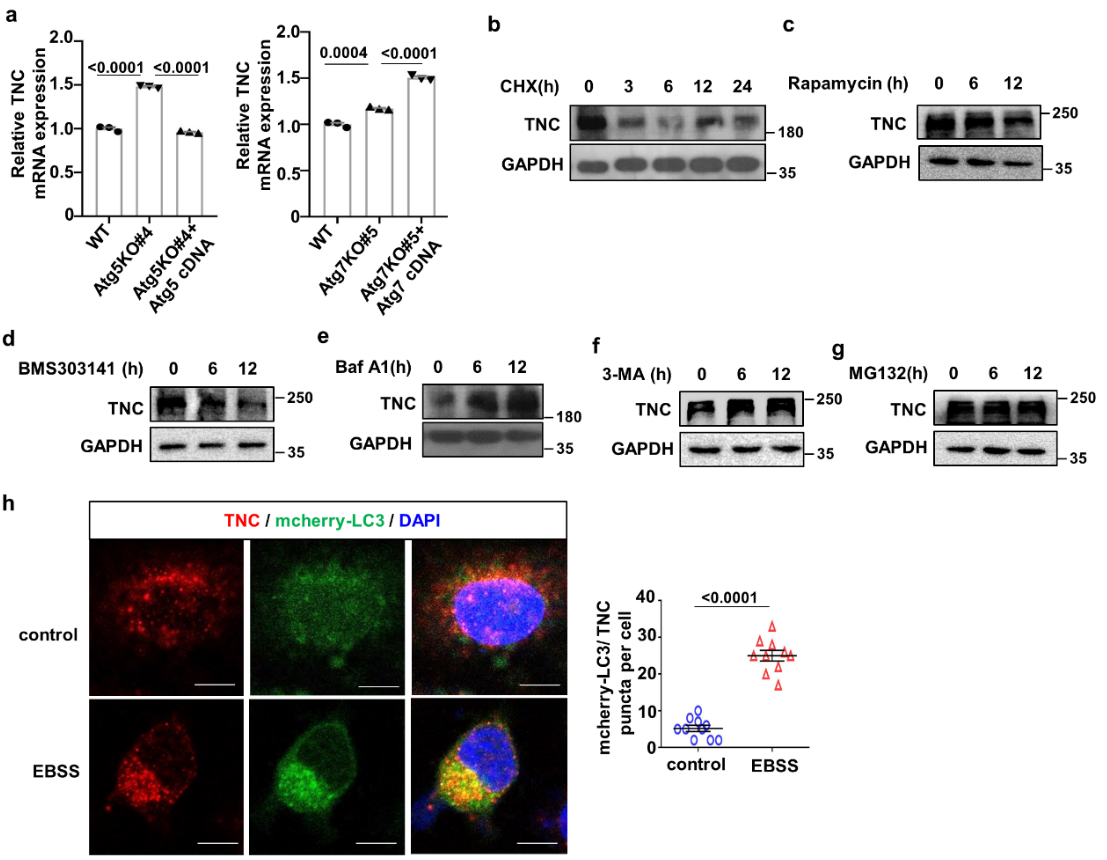

Supplementary Fig. 5 Autophagy selectively degrades TNC via the autophagic receptor p62. (a) The TNC mRNA expression level was detected by qRT-PCR in the indicated MDA-MB-231 cells (n=3 biological independent samples). **(b)** Lysates from MDA-MB-231 cells treated with 20 μM cycloheximide (CHX) at indicated intervals were subjected to immunoblotting. **(c-g)** Immunoblot analysis of extracts of MDA-MB-231 cells treated with 1ug per ml Rapamycin (c), 10uM BS303141(d), 100 nM Baf A1(e), 50uM 3-Methyladenine (3-MA) (f), 10 μM MG132 (g) for the indicated intervals. **(h)** MDA-MB-231 cells were stably transfected with mcherry-LC3 plasmid. Then the cells treated with EBSS in the presence of 100 nM Baf A1 for 3h. Confocal microscopy of the co-localization of mcherry-LC3 with TNC (left). Scar bar, 10um. Statistics shown refer to the puncta formation by mecherry-LC3 with TNC in the indicated samples (n=10 cells per group, right). Error bars represented mean± SEM. The *P* value in **a** was determined by one-way analysis of ANOVA with Tukey's multiple comparisons test, no adjustments were made for multiple comparisons. The *P* value in **h** was determined by a two-tailed unpaired Student's t test. All data are representative of two independent experiments.

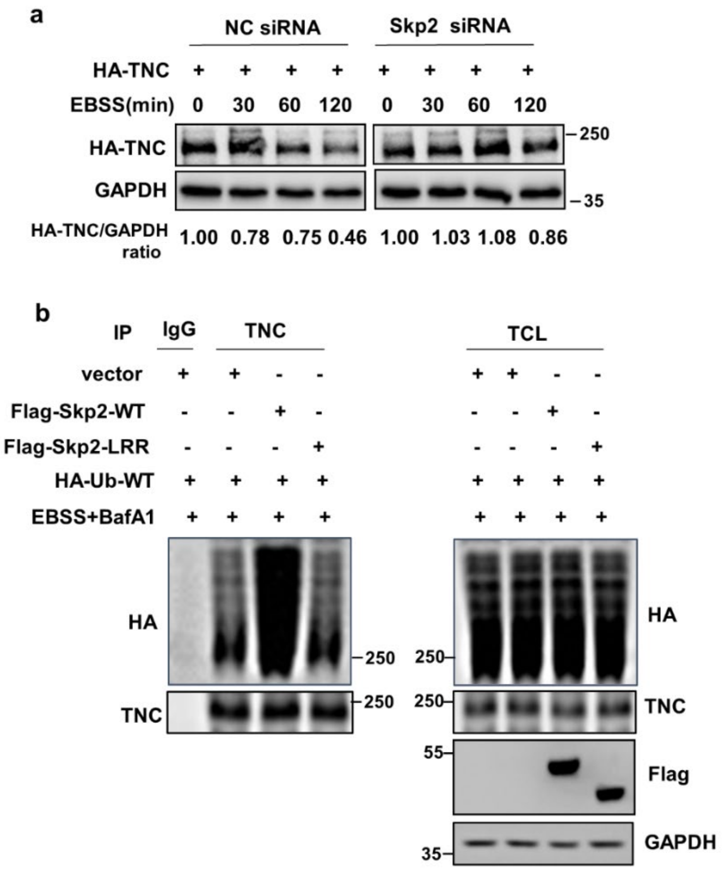

Supplementary Fig. 6 Skp2 is the major E3 ligase ubiquitinating TNC. (a) HEK293T cells were transiently transfected with Skp2 siRNA for 12 h, then co-transfected with HA-tagged TNC for another 48h. Then the cells were treated with EBSS for different hours. **(b)** HEK293T cells were transfected with various combinations of plasmids for 48h, then treated with EBSS treatment in the presence of BafA1 for 2h. Data are representative of three independent experiments.

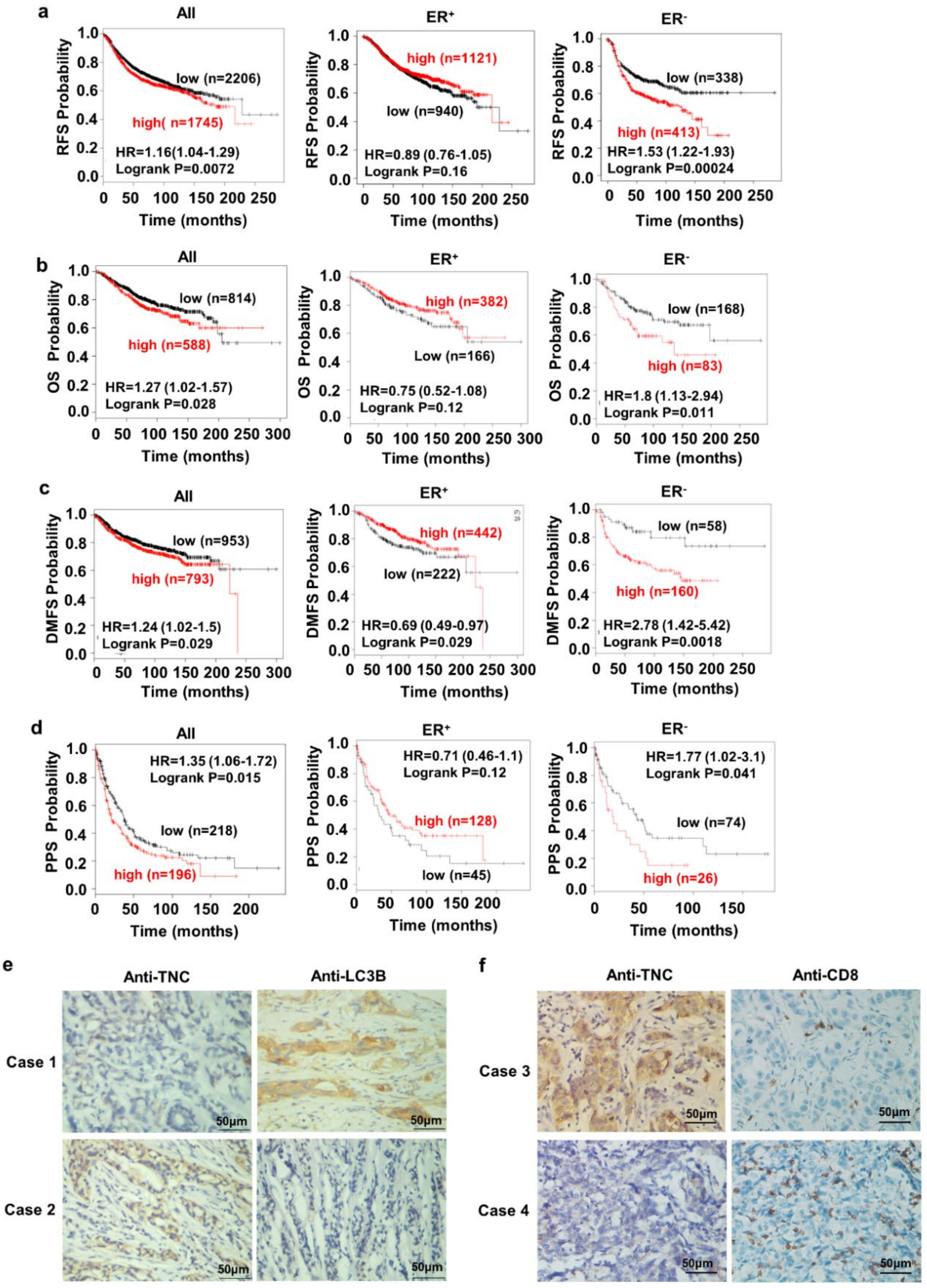

Supplementary Fig. 7 TNC overexpression is associated with poor disease outcome in ER-negative tumours. (a-d) Kaplan-Meier analysis of RFS (a), OS (b), DMFS (c), and PPS (d) respectively, based on TNC (201645_at) mRNA levels using the KM-plotter breast cancer database (http://kmplot.com/analysis). Auto select best cutoff was chosen in the analysis. Patients were stratified according ER status as

indicated. Survival curves were plotted by the Kaplan-Meier method and assessed using the log-rank test, and univariate Cox proportional hazards regression was carried out to identify HR and 95% CI. **(e)** The representative images for LC3B staining in two primary breast tumors with TNC expression. Case 1 showed low expression of TNC expression with positive LC3B staining. Case 2 showed high expression of TNC expression with negative LC3B staining. **(f)** The representative images for CD8 T staining in two primary breast tumors with TNC expression. Case 3 showed high expression of TNC expression with low percentage of tumor-infiltrating $CD8^+$ T. Case 4 showed low expression of TNC expression with high percentage of tumor-infiltrating $CD8^+$ T.

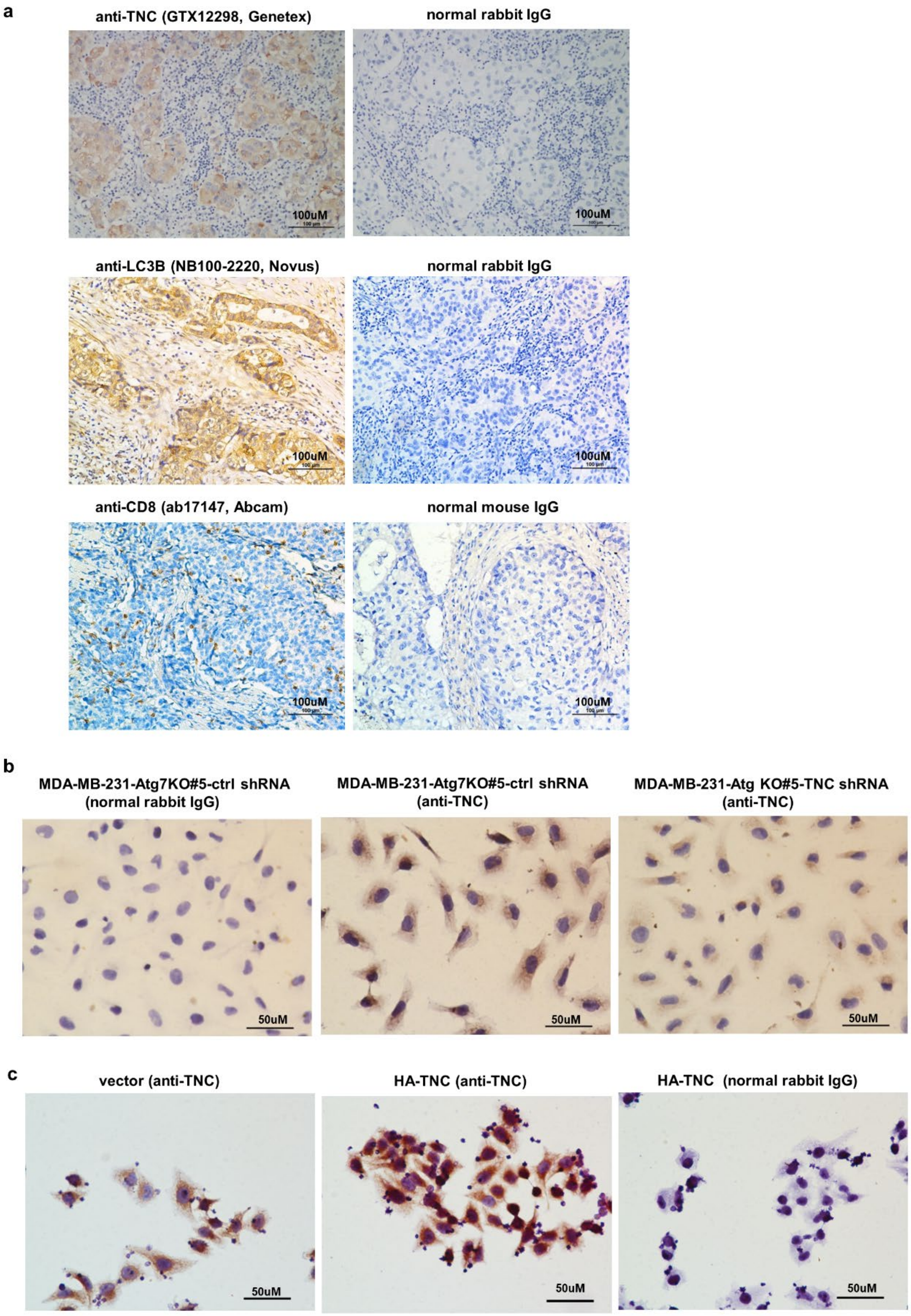

Supplementary Fig. 8 A validation for the specificity of the antibodies. (a) The representative IHC images for TNC, LC3B and CD8 staining in primary TNBC tissues. Normal rabbit and mouse IgG were used as negative controls. **(b)** The representative Immunocytochemistry (ICC) images for TNC staining in the indicated MDA-MB-231

cells. Normal rabbit IgG was used as negative control. **(c)** Hela cells were transiently transfected with HA-tagged TNC plasmids for 48 h. The cells were fixed and stained with anti-TNC for ICC assay. The representative ICC images for TNC staining were showed. Normal rabbit IgG was used as negative control. All data are representative of three independent experiments.

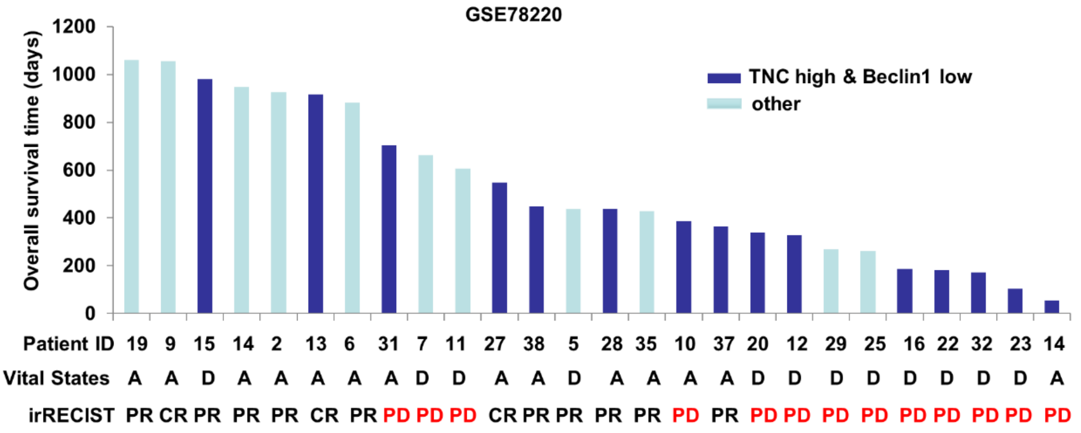

Supplementary Fig. 9 Histogram representation of clinical benefit of anti-PD1 based on TNC and Beclin1 expression. According to their RNA-seq data, 15 patients were classified as "TNC-high and Beclin1-low", while the remaining patients were categorized as "Other". Columns were shaded to indicate TNC and Beclin1 expression status (Vital status: A=alive; D=dead). Immune-related Response Evaluation Criteria in Solid Tumours (irRECIST): CR=complete response; PR=partial response; PD=progression of disease.

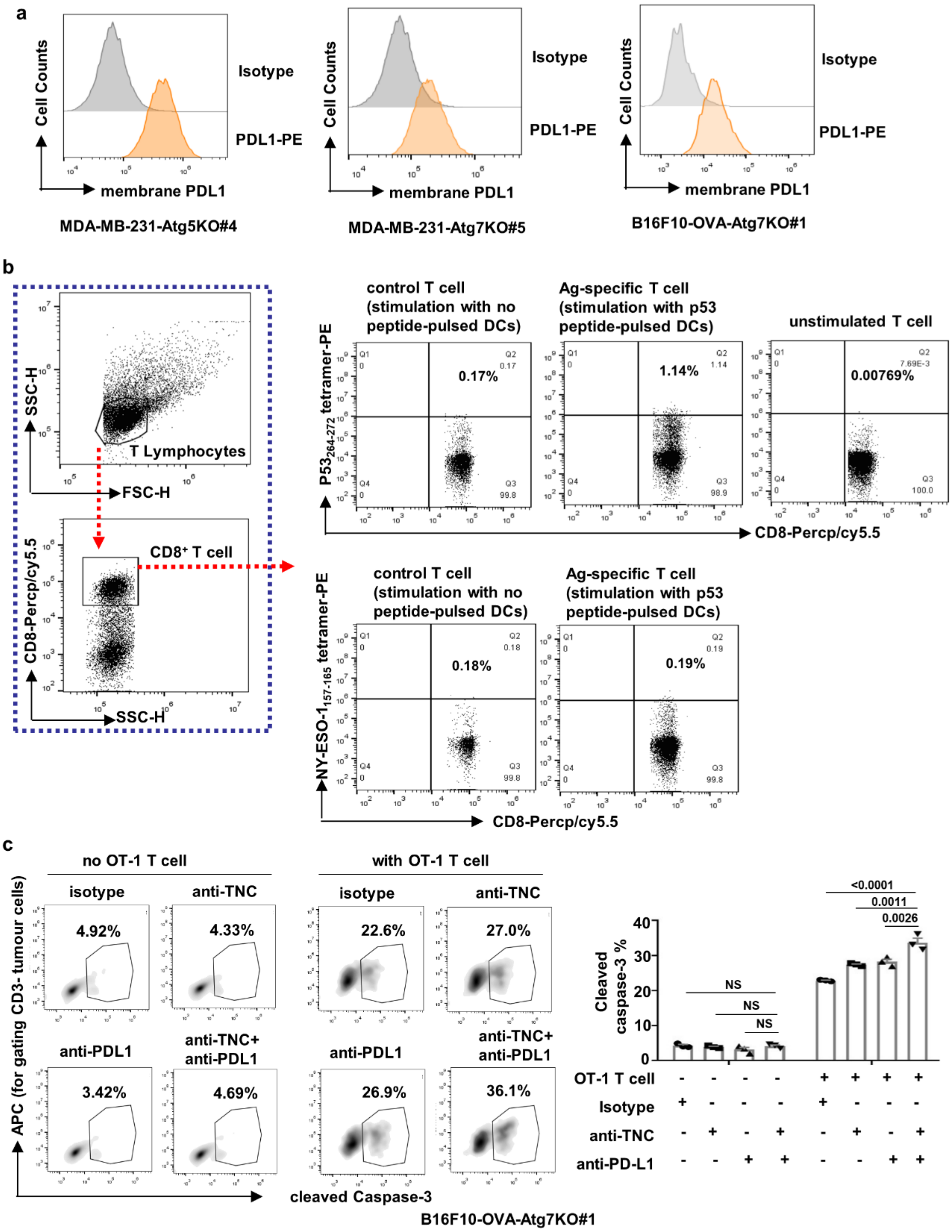

Supplementary Fig. 10 Blockade of TNC sensitizes checkpoint blockade immunotherapy *in vitro.* **(a)** The indicated tumor cells were subjected to FACS analysis for cell surface PDL1-PE expression. **(b)** Autologous purified T cells were cocultured with p53 peptide-pulsed DCs, followed by stained with $p53_{264–272}$ tetramer and anti-CD8. NY-ESO-$1_{157-165}$ tetramer was used as a control tetramer staining. The numbers in the upper right quadrants (Q2) of dot plots indicate the frequency of

$p53_{264\text{-}272}$ tetramer$^+$CD8$^+$ T or NY-ESO-$1_{157\text{-}165}$ tetramer$^+$CD8$^+$ T cells. Unstimulated T cells were from PBMC of the same HLA-A2$^+$ healthy donor. **(c)** After treatment with IFNγ for 24h, B16F10-OVA-Atg7KO#1 cells were pre-treated with anti-TNC (10 ug per ml) or anti-PD-L1 (10ug per ml) for 2 hours, then co-cultured with mouse OT1-CD8$^+$T cells. Left, representative dot plots of the cleavage of caspase-3 in tumour cells measured by flow cytometry. Right, percentage of the cleaved caspase-3 in tumour cells was shown. Error bars represent mean ± SEM, n=3 biological independent samples. The *P* value was determined by one-way ANOVA with Dunnett's multiple comparisons test, no adjustments were made for multiple comparisons. NS, no significance. All data are representative of three independent experiments.

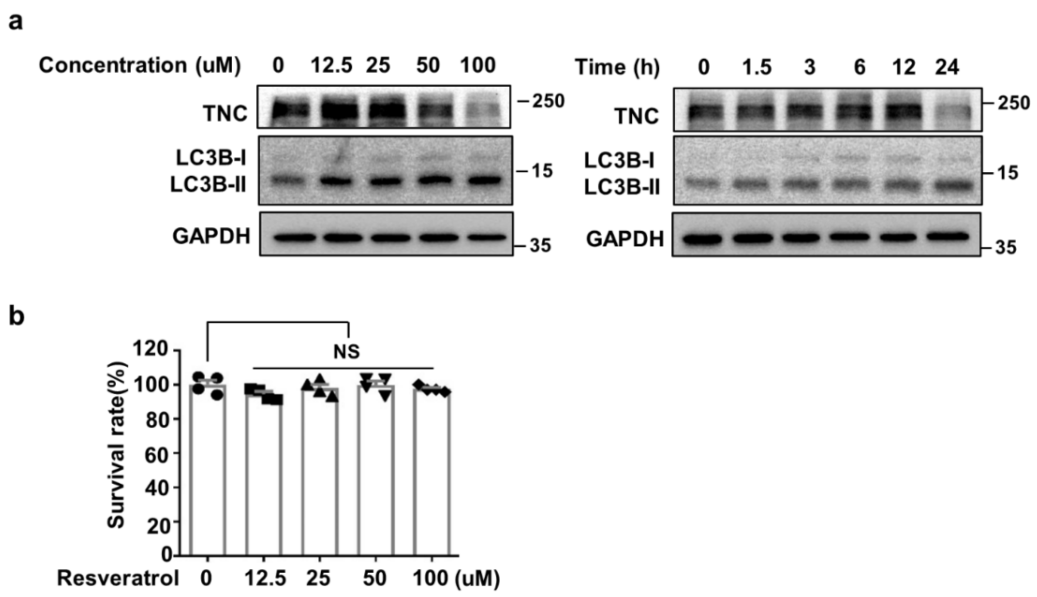

Supplementary Fig. 11 Resveratrol abolished the expression of TNC. (a) Lysates from MDA- MB-231-WT cells treated with various concentrations of compound Resveratrol for 24h or 50uM Resveratrol for the indicated times. **(b)** Growth curves of MDA-MB-231-WT cells were treated by various concentrations of Resveratrol for 24h by MTT assay. Error bars represent mean ± SEM, n=4 biological independent samples. The *P* value in **b** was determined by one-way ANOVA with Dunnett's multiple comparisons test, no adjustments were made for multiple comparisons. NS, no significance. All data are representative of three independent experiments.

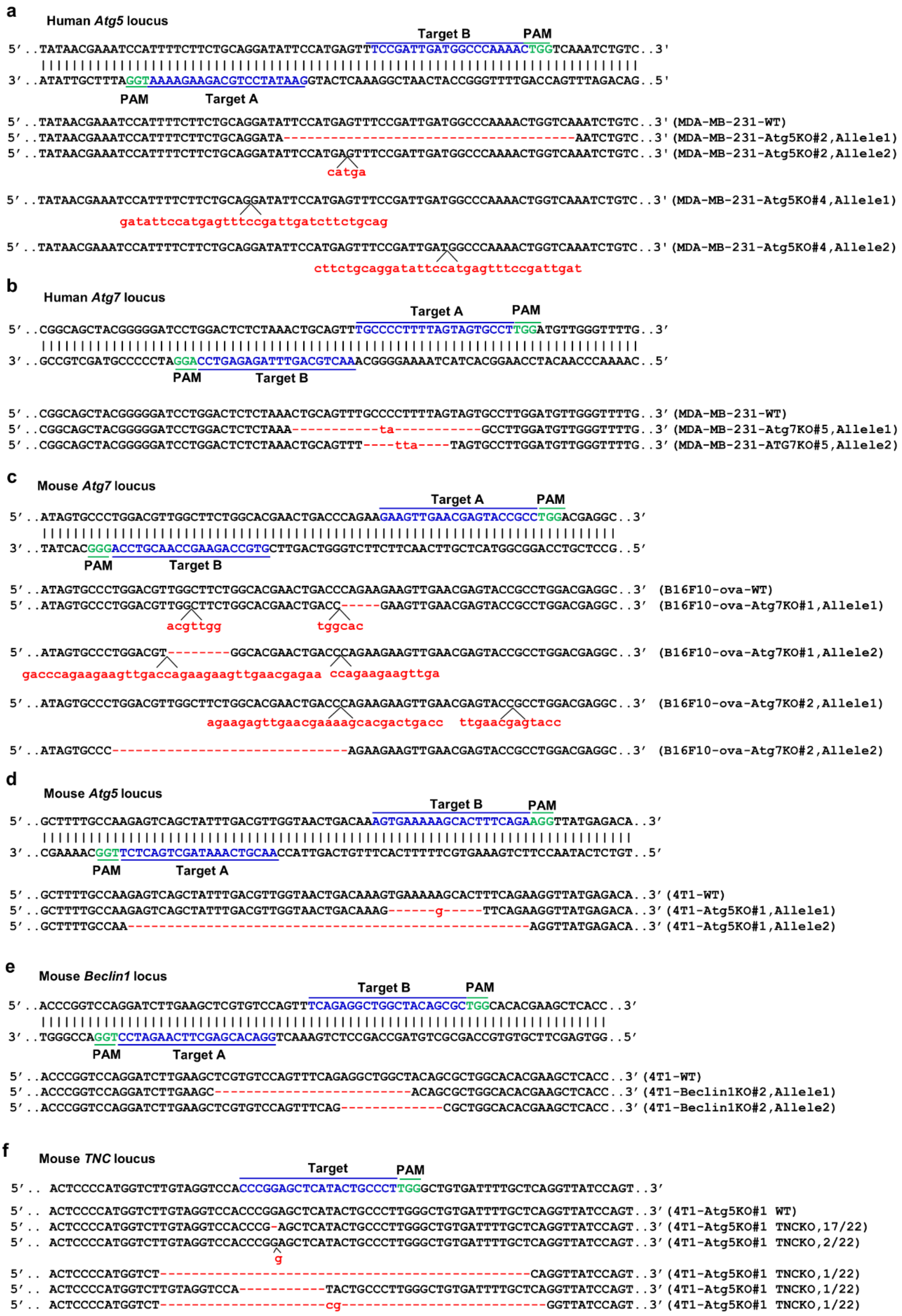

Supplementary Fig. 12 Representative sequences of the Atg5, Atg7, Beclin1 and TNC locus targeted by Cas9n in the indicated cell lines. (a-e) DNA sequencing analysis of targeting ATG5, ATG7, and Beclin1 in the knockout clones include

MDA-MB-231-Atg5KO#2(a), MDA-MB-231-Atg5KO#4(a), MDA-MB-231-Atg7 KO#5(b), B16-OVA-Atg7KO#1(c), B16-OVA-Atg7KO#2(c), 4T1-Atg5KO#1(d), and 4T1-Beclin1KO#2(e). **(f)** DNA sequencing analysis of targeting TNC in 4T1-ATG5KO#1 TNCKO cell line. The PAM sequences are underlined and highlighted in green; the targeting sequences are underlined and highlighted in blue; deletions (-) in red, insertions (^) and mutations, lower case in red; N/N indicates positive colonies out of total sequenced.

Supplementary Table 1. Univariate and multivariate analysis of potential prognostic factors in TNBC breast cancer patients based on overall survival

Factors	Univariate			Multivariate		
	HR	95%CI	*P* value	HR	95%CI	*P* value
Age (> 40 vs. ≤ 40 years)	0.647	0.317～1.321	0.232	-	-	-
T stage (T3-T4 vs. T1-T2)	2.367	1.104～5.076	0.027	1.850	0.845～4.053	0.124
N stage (N2-N3 vs. N0-N1)	2.765	1.391～5.496	0.004	2.555	1.282～5.093	0.008
Grade (3 vs. 1+2)	1.619	0.815～3.217	0.169	-	-	-
TNC (high vs. low)	2.537	1.152～5.586	0.021	2.349	1.063～5.191	0.035
Chemotherapy (Yes vs. No)	1.103	0.389～3.129	0.854	-	-	-

Supplementary Table 2. The detailed information of CRISPR/Cas9 KO plasmids

Targeted genes	Product Cat#	Product Name	Sequences of guide RNA	Targeted region of the genes
Human Atg5	sc-41684 7-NIC	ATG5 Double Nickase Plasmid (h)	Plamid A: GAATATCC TG CAGAAGAAAA<br>Plamid B: TCCGATTG AT GGCCCAAAAC	chr6:106,696,059 -106,696,078<br>chr6:106,696,087 -106,696,106
Mouse Atg5	sc-41914 9-NIC	ATG5 Double Nickase Plasmid (m)	Plamid A: AGTGAAAA AG CACTTTCAGA<br>Plamid B: AACGTCAA AT AGCTGACTCT	chr10:44,289,907 -44,289,926<br>chr10:44,289,875 -44,289,894
Human Atg7	sc-40099 7-NIC	ATG7 Double Nickase Plasmid (h)	Plamid A: TGCCCCTTT TA GTAGTGCCT<br>Plamid B: AACTGCAG TT TAGAGAGTCC	chr3:11,340,214-11,340,233<br>chr3:11,340,194-11,340,213
Mouse Atg7	sc-42880 5-NIC	ATG7 Double Nickase Plasmid (m)	Plamid A: GAAGTTGA AC GAGTACCGCC<br>Plamid B: GTGCCAGA AG CCAACGTCCA	chr6:114,673,079 -114,673,098<br>chr6:114,673,045 -114,673,064
Mouse Beclin1	sc-42503 3-NIC	BECN1 Double Nickase Plasmid (m)	Plamid A: GGACACGA GC TTCAAGATCC<br>Plamid B: TCAGAGGC TG GCTACAGCGC	chr11:101,301,758-101,301,777<br>chr11:101,301,782-101,301,801

Supplementary Table 3. Antibodies used for flow cytometry analysis

Antibody	Company	Catalog number
FITC Anti-Active Caspase-3	BD Bioscience	559341
PE/Dazzle™ 594 anti-mouse CD4	Biolegend	100455
Ms IFN-Gma Alexa 647 XMG1.2	BD Bioscience	557735
PerCP/Cy5.5 anti-mouse CD8a	Biolegend	100733
PE/Cy7 anti-mouse CD274	Biolegend	124313
APC/Cy7 anti-mouse CD45	Biolegend	103116
APC anti-mouse CD3	Biolegend	100235
FITC anti-human HLA-A2	Biolegend	343303
PE anti-human CD274	eBioscience	12-5983-42
APC anti-human CD3	Biolegend	300412
PE anti-human HLA-ABC	eBioscienc	12-9983-42
PerCP/Cy5.5 anti-human CD8a	Biolegned	344710
APC anti-human CD4	Tonbo Biosciences	20-0049-T100

Supplementary Table 4. Antibodies used in immunoblot and immunoprecipitation

Antibody	Company	Catalog number
TNC	Cell Signaling Technology	12221
TNC	Santa Cruz	20932
Beclin1	Cell Signaling Technology	3738
P62	Santa Cruz	sc-28359
Atg5	Cell Signaling Technology	12994
Atg7	Cell Signaling Technology	2631
Atg3	Cell Signaling Technology	3415
Atg10	Abgent	AP14315
Atg13	Cell Signaling Technology	13468
Atg14	Cell Signaling Technology	96752S
uvrag	Abgent	AP1850b
VPS34	Cell Signaling Technology	4263s
HA	Cell Signaling Technology	2367
HIF-1α	Cell Signaling Technology	14179
β-TRCP	Santa Cruz Biotechnology	sc-33213
LC3B	Novus	NB100-2220
GAPDH	Cell Signaling Technology	5174
TRAF6	Abcam	ab33915
skp2	Cell Signaling Technology	4358
cdh1	CALBIOCHEM	CC43
Flag	Sigma	F1804
Flag	Cell Signaling Technology	2368S
Myc-tag	Cell Signaling Technology	2276
CRISPR-Cas9	Abcam	Ab191468
P53	Cell Signaling Technology	2524
β-actin	Santa Cruz	sc-130656
Anti-mouse IgG, HRP-linked	Cell Signaling Technology	7076
Anti-rabbit IgG, HRP-linked	Cell Signaling Technology	7074

Supplementary Table 5. Oligonucleotide sequence of siRNAs

Gene Name	Sequence
β-TrCP siRNA(human)	5'-AAGUGGAAUUUGUGGAACAUC-3'
Skp2 siRNA (human)	5'-GCUUCACGUGGGGAUGGGA-3'
Cdh1 siRNA (human)	5'-UGAGAAGUCUCCCAGUCAG-3'
Atg7 siRNA(human)	5'-GGGUUAUUACUACAAUGGUGUU-3'
Beclin1 siRNA(human)	5'-GAUUGAAGACACAGGAGGCTT-3'
Atg3 siRNA(human):	5'-GGGAAAGGCACUGGAAGUG-3'
Atg10 siRNA(human)	5'-GGAGUUCAUGAGUGCUAUA-3'
Atg13 siRNA(human)	5'-GAGUUUGGAUAUACCCUUU-3'
Atg14 siRNA(human)	5’-CCGGGAGAGGUUUAUCGACAAGATT-3’
Uvrag siRNA(human)	5’-GCCAGACCGUCUUGAUACA-3’
VPS34 siRNA(human)	5’-GUUGAAGUUCUCAGGACUAUATT-3’
Atg5 siRNA(human)	5'-GCAACTCTGGATGGGATTG-3'
P62 siRNA(human)	5'-GCAUUGAAGUUGAUAUCGAUU-3'

Supplementary Table 6. Antibodies used in IHC

Antibody	Company	Catalog number
TNC	Genetex	GTX12298
LC3B	Novus	NB100-2220
CD8	Abcam	ab17147
CD4	Abcam	ab183685
granzyme B	Abcam	ab4059
Normal mouse IgG	Merck millipore	12-371
Normal rabbit IgG	Cell Signaling Technology	2729S

Supplementary References

1. Mai J*, et al.* Polo-Like Kinase 1 phosphorylates and stabilizes KLF4 to promote tumorigenesis in nasopharyngeal carcinoma. *Theranostics* **9**, 3541-3554 (2019).

2. Hoos A, Wolchok JD, Humphrey RW, Hodi FS. CCR 20th Anniversary Commentary: Immune-Related Response Criteria--Capturing Clinical Activity in Immuno-Oncology. *Clinical cancer research : an official journal of the American Association for Cancer Research* **21**, 4989-4991 (2015).
