# Supplementary material for: Autophagy deficiency promotes triple-negative breast cancer resistance to T cell-mediated cytotoxicity by blocking tenascin-C degradation
Source: Nat Commun. 2020 Jul 30;11:3806. doi: 10.1038/s41467-020-17395-y (PMC7393512; doi:10.1038/s41467-020-17395-y)
Supplement: Supplementary file 3 — Description of Additional Supplementary Files [file 41467_2020_17395_MOESM3_ESM.pdf]

Description of Additional Supplementary Files

File Name: Supplementary Data 1
Description: MS identified quantification details for MDA-MB-231 WT and MDA-MB -231 Atg5 KO#4 using a SILAC assay (provided as an Excel file).

File Name: Supplementary Data 2
Description: MS identified quantification details for MEF WT and MEF Atg5 -/- using a SILAC assay (provided as an Excel file).
